# Cross Talk Between Cyclic Nucleotides and Calcium Signaling Pathways in Plants–Achievements and Prospects

**DOI:** 10.3389/fpls.2021.643560

**Published:** 2021-02-16

**Authors:** Brygida Świeżawska-Boniecka, Maria Duszyn, Mateusz Kwiatkowski, Adriana Szmidt-Jaworska, Krzysztof Jaworski

**Affiliations:** Chair of Plant Physiology and Biotechnology, Faculty of Biological and Veterinary Sciences, Nicolaus Copernicus University in Toruń, Toruń, Poland

**Keywords:** cAMP, cGMP, calcium, plant signaling, plant cell

## Abstract

A variety of plant cellular activities are regulated through mechanisms controlling the level of signal molecules, such as cyclic nucleotides (cNMPs, e.g., cyclic adenosine 3′:5′-monophosphate, cAMP, and cyclic guanosine 3′:5′- monophosphate, cGMP) and calcium ions (Ca2^+^). The mechanism regulating cNMP levels affects their synthesis, degradation, efflux and cellular distribution. Many transporters and the spatiotemporal pattern of calcium signals, which are transduced by multiple, tunable and often strategically positioned Ca2^+^-sensing elements, play roles in calcium homeostasis. Earlier studies have demonstrated that while cNMPs and Ca2^+^ can act separately in independent transduction pathways, they can interact and function together. Regardless of the context, the balance between Ca2^+^ and cNMP is the most important consideration. This balance seems to be crucial for effectors, such as phosphodiesterases, cyclic nucleotide gated channels and cyclase activity. Currently, a wide range of molecular biology techniques enable thorough analyses of cellular cross talk. In recent years, data have indicated relationships between calcium ions and cyclic nucleotides in mechanisms regulating specific signaling pathways. The purpose of this study is to summarize the current knowledge on nucleotide-calcium cross talk in plants.

## Introduction

**FIGURE 1:**
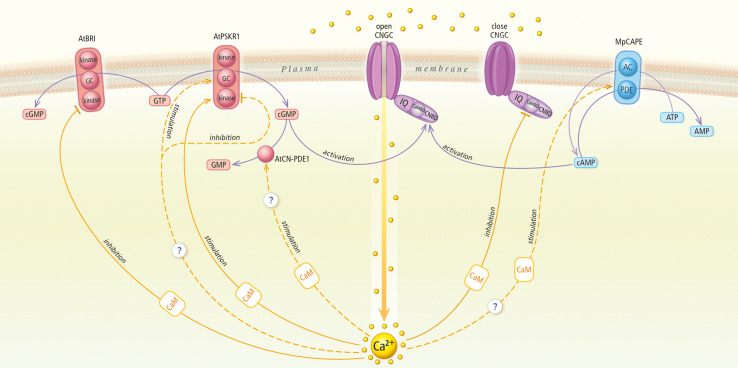
Cyclic nucleotides and calcium signaling pathways in plant cells. The figure draws on actual knowledge about cyclic nucleotide-calcium cross talk in plants. AtBRI1, *A. thaliana* brassinosteroid receptor; GC, guanylyl cyclase; cGMP, cyclic guanosine monophosphate; GTP, guanosine triphosphate; AtPSKR1, *A. thaliana* phytosulfokine receptor 1; AtCN-PDE, *A. thaliana* cGMP-activated phosphodiesterase; GMP, guanosine monophosphate; CNGC, cyclic nucleotide gated channel; CNBD, cyclic nucleotide binding domain; CaMBD, calmodulin-binding domain; IQ, isoleucine-glutamine motif; MpCAPE, M. polymorpha combined AC with PDE; AC, adenylyl cyclase; PDE, phosphodiesterase; AMP, adenosine monophosphate; cAMP, cyclic adenosine monophosphate; ATP, adenosine triphosphate; CaM, calmodulin.

Substantial evidence has indicated that calcium ions (Ca^2+^) and cyclic nucleotides (cAMP/cGMP) cooperate in many (pato)physiological processes in plants. Both of these signaling pathways form a complex network with some control points. The interaction between calcium and cyclic nucleotides was observed in the protoplasts of *Nicotiana plumbaginifolia* (Volotovski et al., 1998), guard cells of *Arabidopsis thaliana* and *Vicia faba* (Curvetto et al., 1994; Lemtiri-Chlieh and Berkowitz, 2004) and pollen tubes of *Pyrus pyrifolia* (Wu et al., 2011). These reports are mainly focused on cyclic nucleotide gated channels (CNGCs), which are junctions of cyclic nucleotides and Ca^2+^ signaling pathways and comprise the most extensively studied group of cNMP targets (Ali et al., 2007; Duszyn et al., 2019). However, some recent reports have shown evidence or strongly suggested that calcium may

interact with other targets of cyclic nucleotides, such cNMP phosphodiesterases (PDEs) or adenylyl/guanylyl cyclases (ACs/GCs) (Muleya et al., 2014; Kwezi et al., 2018; Figure 1). cAMP and cGMP pathways apparently can be modified by calcium ions alone and/or calcium effectors, such as calmodulin (CaM), at different levels.

The purpose of this mini review is to provide an update on our understanding of calcium and cyclic nucleotide signaling integration. Specifically, we highlight the regulatory effect of Ca^2+^ ions on enzymes involved in cNMP metabolism, i.e., cyclases, which are critical for cyclic nucleotide synthesis, and phosphodiesterases, which are critical for its inactivation.

## Cooperation of Ca^2+^ and Adenylyl/Guanylyl Cyclases

Adenylyl/guanylyl cyclases catalyze the formation of 3′-5′-cyclic adenosine/guanosine monophosphate (cAMP/cGMP) from adenosine and guanosine-5′-triphosphate (ATP/GTP), respectively. Recently, evidence has shown that the activity of plant transmembrane GCs is modulated by calcium ions (Oh et al., 2012; Muleya et al., 2014). Bioinformatics research has indicated that GCs possess potential calmodulin (CaM)-binding sites or calcium-binding EGF-like (Ca-EGFL) domains within the GC domain (Meier et al., 2010; Oh et al., 2012; Hartmann et al., 2014). The regulatory mechanism of calcium ions on a group of plant kinases called “moonlighting” proteins, in which a functional GC domain is embedded within an intracellular kinase domain, was studied and confirmed (Irving et al., 2012; Oh et al., 2012; Muleya et al., 2014). Many of these “moonlighting” proteins contain dominant kinase function and an additional cyclase function (Wong et al., 2015). The spatial arrangement of these two domains is somewhat unexpected and since both enzymatic activities are regulated by molecules such as calcium and the catalytic product of each domain, these proteins are likely to serve as molecular tuners (Irving et al., 2018). However, to date, no direct calcium ion fixation sites have been detected within plant GCs. *In vitro* experiments have shown that the GC activity of the phytosulfokine receptor AtPSKR1, one of the “moonlighting” proteins from *A. thaliana*, is significantly enhanced by calcium at physiological levels (0.1–10 μM) (Muleya et al., 2014). More importantly, the same concentration of Ca^2+^ that activates the cyclase domain also exerts an inhibitory effect on kinase activity. Therefore, calcium ions have a direct but antagonistic effect on the alternate intrinsic activities of PSKR1. The authors rightly suggested that calcium ions can act as molecular switches of the guanylyl/kinase activity of select plant proteins (Muleya et al., 2014). However, the stimulating effect of Ca^2+^ on GC activity has only been analyzed by *in vitro* biochemical techniques. This outcome led to the initial conclusion that the local concentration of cytosolic calcium ions caused by receptor activation is the key regulatory factor in the dual GC and kinase activities of this bifunctional protein. The mechanism of calcium ions and plant GCs cooperation needs to be elucidated in detail because the molecular basis of this regulation remains a mystery.

The knowledge that calcium ions can affect plant kinase activity is not new (Cheng et al., 2002; Valmonte et al., 2014). The novelty and surprise stems from the discovery that calcium ions can activate GCs. Animal GCs usually interact with components in the G protein cascade, which is activated by low levels of calcium (Kobiałka and Gorczyca, 2000). However, this signaling pathway has not been confirmed in plants. Therefore, plant cyclases presumably must bind Ca^2+^ directly or indirectly via intermediary proteins. Bioinformatics and *in vitro* analyses revealed that a functional calmodulin-binding motif is localized within the kinase domain of AtPSKR1 (Hartmann et al., 2014). An interaction between CaM and AtPSKR1 was analyzed and showed that AtPSKR1 can bind to all isoforms of calmodulin. It was thus suggested that calcium can stimulate the kinase activity of PSKR1 via Ca^2+/^CaM calmodulin isoforms, however, the effect of this interaction on the PSKR1 GC domain activity was not analyzed by the authors.

Another bifunctional protein with both guanylyl cyclase and kinase domains is the brassinosteroid receptor from *A. thaliana* (AtBRI) (Kwezi et al., 2007; Wheeler et al., 2017). It is reportedly a binding target of CaM, similar to AtPSKR1 (Oh et al., 2012). Protein-interaction studies revealed that the kinase domain of the AtBRI1 can interact with calmodulin *in vitro*, and the specific interaction was observed in the presence of only calcium ions. Calmodulin binding inhibited BRI kinase activity, in contrast to PSKR1. This finding suggests that autophosphorylation may be modulated by CaM in a more specific manner. For instance, the tyrosine kinase activity of AtBRI1 was more sensitive to CaM than the serine/threonine kinase activity of AtPSKR1. However, experiments showing the influence of calcium ions on the GC activity of AtBRI1 have not been performed. Additionally, the GC activity of this protein is debated because studies determining its crystal did not confirm this activity (Bojar et al., 2014).

The available data show that the effect of calcium ions on cyclase activity occurs indirectly via CaM isoforms. However, an exception is described in *A. thaliana*: wall-associated kinase-like 10 (AtWAKL10), a protein of the “moonlighting” group with GC activity that contains an extracellular calcium-binding EGF-like domain and a degenerate EGF2-like domain, motifs that directly bind Ca^2+^ (Verica and He, 2002; Meier et al., 2010). The authors suggest that AtWAKL10 may sense changes in apoplastic calcium concentrations and cell wall pectin composition generated in response to pathogen attack and transmit a signal to the cytoplasm via its intracellular kinase or GC domain. Additionally, a correlation of the high expression of *AtWAKL10* with genes encoding calcium transporting and sensing proteins was revealed (Meier et al., 2010).

Interestingly, GCs are discussed as potential common points of cGMP and nitric oxide (NO), signaling pathways (Marondedze et al., 2015; Gross and Durner, 2016). Bioinformatics analysis revealed that some of plant GCs possess highly conserved heme-NO and oxygen−binding (H-NOX) domains (Mulaudzi et al., 2011; Gehring and Turek, 2017). Recent reports showed that diacylglycerol kinase 4 (DGK4) from *A. thaliana* contains the GC domain modulating NO responses (Vaz Dias et al., 2019; Angkawijaya et al., 2020; Wong et al., 2020). The authors confirmed that DGK4 is a link between NO, cNMP and Ca^2+^ signaling in pollen tube guidance.

Some data show plant GC interactions with calcium ions, however, as in the case of ACs, no experimentally obtained information has confirmed a regulatory effect. In contrast to plant proteins, the direct and indirect modulation of animal adenylyl cyclase activity by Ca^2+^/CaM and Ca^2+^ has been documented (Halls and Cooper, 2011). A bioinformatics analysis of amino acid sequences^1^ revealed that calmodulin-binding sites are localized in plant proteins such as AtKUP5 and AtKUP7 in the C-terminus (Al-Younis et al., 2015, 2018) and AtClAP in the N-terminus (Chatukuta et al., 2018), potentially acting as ACs. However, these bioinformatics predictions should be checked experimentally.

The above mentioned knowledge may suggest only that cNMP cyclases can among the cross points of cyclic nucleotides and calcium pathways, however, determining the mechanism of the regulatory effects remains a challenge.

## Cooperation of Ca^2+^ and cNMP Phosphodiesterases (cNMP PDEs)

Phosphodiesterases are enzymes that terminate the action of cyclic nucleotides by their inactivation to 5′- and 3′-nucleotide monophosphates via the hydrolysis of their 3′-phosphoester bonds (Gross and Durner, 2016). Phosphodiesterases comprise a large family of enzymes that can be categorized into appropriate groups based on their regulatory/kinetic properties: Ca^2+^/calmodulin stimulated, cGMP stimulated, cGMP inhibited – cAMP selective and cAMP stimulated (Essayan, 2001). The research to date is marginal but very important for understanding the mechanism by which transient cNMP concentrations are regulated. Only two cases have been molecularly confirmed thus far: phosphodiesterase coupled with the adenylyl cyclase domain (MpCAPE) in liverwort (*Marchantia polymorpha*) (Kasahara et al., 2016) and cGMP-activated phosphodiesterase (AtCN-PDE1) in *A. thaliana* (Isner et al., 2019). Bioinformatics tools revealed calmodulin-binding sites in both PDEs, suggesting that calcium may be a necessary intermediate element in the cNMP inactivation pathway in plants.

Calcium ions/calmodulin-stimulated PDEs comprise a group of enzymes widely distributed in the superkingdom of eukaryotes. Their activation is the result of the attachment of calmodulin activated by Ca^2+^ (Kakkar et al., 1999; Sharma, 2003). Why do many types of PDEs that have a regulatory mechanism in which the presence of CaM is not needed for their activation have high affinity calmodulin-binding sites within their catalytic domains? For example, PDE3A has an NHR regulatory region and PDE5A and PDE10A each have a GAF regulatory domain (Ahmad et al., 2015). A situation that is potentially related to that of plant PDEs has been observed. Both described enzymes have CaM-binding sites, but they are active even in the absence of a CaM/Ca^2+^ complex in the reaction buffer. Another discovery revealed that in plant PDEs, the CaM-binding sites are located within or just outside catalytic domains, in contrast to animal PDEs, where the domain is in the N-terminus (Conti and Beavo, 2007). This finding suggests that CaM can act as an additional, indirect PDE stimulator activated by cNMP to stimulate calcium channels. The effect of plant calmodulin on PDE in cattle has been tested, and the results showed that two soya isoforms affected PDE activity in different ways because they had different Ca^2+^ requirements (Lee et al., 2000).

A protein group called CAPE have been identified in *Streptophyta* and consists of “moonlighting” proteins with PDE and AC domains (Kasahara et al., 2016). Despite their similar structures, the orthologs of these proteins found in plants have different numbers of calmodulin-binding sites. However, the roles of these proteins in plant cells, as well as their regulation and interaction mechanisms, are unknown. CAPE proteins are present in antheridium - haploid structures that produce and contain male gametes, where Ca^2+^ is required for the formation and differentiation of antheridia (Ruth et al., 1988). Recent studies have also shown the expression of genes encoding cAMP-dependent kinase and cyclic nucleotide-gated ion channels in the antheridium of *M. polymorpha* (Higo et al., 2016). These data indicate that effectors regulated by Ca^2+^ and cNMP can work together in antheridium formation and spermatogenesis. The likelihood of this regulatory interaction may be true because cAMP in animal cells is an irreplaceable factor in spermatogenesis and sperm physiology (Buffone et al., 2014).

Phosphodiesterases from various groups of animals can be phosphorylated and activated by PKA and PKG, but in the case of CaM-dependent PDEs, the phosphorylation process decreases the affinity of CaM to PDE, which results in its inactivation (Keravis and Lugnier, 2010). If the cNMP signaling pathway in plants has not evolved differently than it did in animals, calcium can be assumed to modulate PDE activity indirectly through its impact on plant kinases, especially since the presence of PKG in plants has recently been molecularly confirmed (Shen et al., 2019). Phosphorylation of N-terminal domains causes a change in the PDE catalytic domain conformation with an increase in V_max_ or a decrease in K_m_ toward the substrate and may also modify its interaction with inhibitors (Conti and Beavo, 2007). However, notably, only the presence of these kinases has been molecularly confirmed, and an examination of newly discovered plant PDEs in terms of the regulation of their activity will indicate whether calcium is an important element in the pathway of cyclic nucleotide degradation.

## Cooperation Between Ca^2+^ and CNGCs

Cyclic nucleotide-gated channels (CNGCs) comprise a family of channels involved in the uptake and transport of Ca^2+^, and their regulation depends on the binding of cNMP. Plant CNGCs have a complex structure composed of six transmembrane helices and pore region or P-loop. Moreover, CNGCs contain overlapping cyclic nucleotide-binding domains (CNBDs) and calmodulin-binding domains (CaMBDs). In the C-terminus of some CNGCs, the IQ motif (isoleucine-glutamine motif) has been identified. It is an additional domain through which CNGCs can interact with CaMs (Zelman et al., 2012, 2013; Duszyn et al., 2019).

First, it seemed that channel activity is regulated by the reversible binding of cyclic nucleotides or calmodulin. The proposed model was based on channel characteristics, overlapping binding sites for cNMPs and CaM. Specifically, Ca^2+^-CaM binding to the C-terminus displaces the cyclic nucleotide from its binding site, leading to channel closure (Kaplan et al., 2007). However, later findings showed that CNGCs may contain two alternative CaM-binding motifs, CaMBD and IQ, suggesting more complicated regulation (Fischer et al., 2013). A model of CNGC regulation by CaM has been proposed in which calcium-free calmodulin is permanently anchored to the IQ domain. This interaction allows calmodulin to function as a precise Ca^2+^ sensor of the channel complex, providing Ca^2+^-dependent feedback (Fischer et al., 2017). Moreover, the IQ motif is highly conserved in most CNGC families, not only in flowering plants but also in distantly related non-vascular *P. patens*. The existence of this domain can be associated with the function of CNGCs, and it has great importance and shows the sophisticated significance of CaM in plant CNGC regulation. Subsequent experiments seem to confirm this model by revealing the relationship between various *A. thaliana* calmodulins and channels (Pan et al., 2019; Tian et al., 2019; Zeb et al., 2020). However, further work is needed to explain why calmodulin, particularly specific isoforms, is essential for the functioning of a specific channel and whether the binding of calmodulin elicits an identical reaction in all channels. Considering the functional range of CNGCs in plant growth, development and the stress response, multiple CaM-binding sites may be required for the up- or downregulation of specific channels.

Cyclic nucleotide gated channels constitute a group of non-specific, voltage-gated cation channels with varying degrees of ion conduction selectivity. The first report on plant CNGC indicated that AtCNGC2 facilitates cyclic nucleotide-dependent cation currents (Leng et al., 1999). More recent analyses showed that AtCNGC2 mediates Ca^2+^ influx in leaf cells. Moreover, disrupting channel activity caused an accumulation of a significant amount of Ca^2+^ in the extracellular space of leaf cells, indicating the importance of plant cells maintaining low extracellular Ca^2+^ levels through AtCNGC2-mediated deposition of Ca^2+^ (Wang et al., 2017). Rahman et al. (2020) proposed a model for cGMP signal transduction in the resistance of tomato to diverse pathogens. Specifically, it suggests that pathogen-related stimuli are recognized by specific receptors that transduce signals to GCs embedded within kinases, which consequently leads to cGMP accumulation and CNGC-dependent activation, which results in cytosolic Ca^2+^ elevation. A similar relationship between cyclases and CNGCs was observed in other studies, linking the immune signaling facilitated by guanylyl cyclase (AtPepR1) to the function of a cGMP-activated Ca^2+^-conducting channel — AtCNGC2 (Qi et al., 2010). Although the roles of cyclic nucleotides in CNGC regulation seemed certain (Leng et al., 2002; Ali et al., 2007; Wang et al., 2013), recent reports showed that activation of AtCNGC11 and AtCNGC12 was not affected by cNMPs (Zhang et al., 2019). These findings suggest that not all plant CNGCs are gated by cyclic nucleotides. This is significantly different from their animal counterparts, which are allosterically regulated when the cNMPs interact with the CNBD. In plants, the binding affinities of cAMP and cGMP for CNBDs have not been determined. Therefore, the precise roles of cNMPs in channel opening and their functions remain unclear. Further research is necessary to resolve these controversial reports and determine whether CNGCs are truly gated by cyclic nucleotides. It is worth verifying whether channel regulation is more complex and requires an additional factor not yet discovered. To date, calcium and calmodulin seem to be the main molecules regulating CNGC activity. Subsequent analyses will provide a better understanding of the roles of cNMP, Ca^2+^, and CaM in CNGC regulation and cooperation.

## Conclusion and Future Perspectives

The plant cyclic nucleotide signaling pathway have a complex evolutionary history that cannot simply be inferred from taxonomic and phylogenetic relationships.

The existence of conserved and divergent features revealed by always updated genomic data is a useful tool to address this topic and clarify the origin and current role of these important signaling molecules. Functional “systemic” analysis are in demand to study the cNMP and Ca2^+^ signaling cross-talk network to validate *in vivo* all the stimulatory and inhibitory regulatory functions depicted in Figure 1. These actions are mediated by the dynamics of two cyclic nucleotides: cAMP and cGMP, as well as the appearance or disappearance of Ca^2+^. For many years, we have been interested in determining the relationship between calcium and cyclic NMP in the regulation of cNMP biosynthesis, function and inactivation. Accumulated knowledge indicates that strictly regulated relationships are the basis of plant growth and development processes, which was observed in physiological experiments. However, the level of cooperation or mechanism by which Ca^2+^ and cNMP signals integrate into genetic pathways that regulate processes remain unknown. Data confirm that the activity of signaling pathways is selectively regulated by the cross-talk between Ca^2+^ and cNMP signaling components. These messengers can interact at multiple sites on at least two levels, the metabolic level, e.g., both cNMP metabolism and Ca^2+^ transport, and the functional level, e.g., cellular processes regulated by one messenger can often be modulated by the other. The high degree of complexity in this interactive system provides the cell with the flexibility required for producing a wide range of specific responses. Future challenges include clarification of the antagonistic and agonistic interactions between the Ca^2+^- and cAMP/cGMP-derived signals in a spatiotemporal manner at the molecular level, later events of signaling maintenance, and the links between these interactions and events to other important transcriptional regulatory networks.

## Author Contributions

BŚ-B, MD, and MK wrote the manuscript. AS-J and KJ revised and critically evaluated the manuscript. All authors read and approved the manuscript.

## Conflict of Interest

The authors declare that the research was conducted in the absence of any commercial or financial relationships that could be construed as a potential conflict of interest.

## Footnotes

1 http://calcium.uhnres.utoronto.ca/ctdb/ctdb/sequence.html
